# An Introductory Framework for Choosing Spatiotemporal Analytical Tools in Population-Level Eco-Epidemiological Research

**DOI:** 10.3389/fvets.2020.00339

**Published:** 2020-07-07

**Authors:** Kaushi S. T. Kanankege, Julio Alvarez, Lin Zhang, Andres M. Perez

**Affiliations:** ^1^Department of Veterinary Population Medicine, College of Veterinary Medicine, University of Minnesota, Saint Paul, MN, United States; ^2^Departamento de Sanidad Animal, Centro de Vigilancia Sanitaria Veterinaria (VISAVET), Facultad de Veterinaria, Universidad Complutense, Madrid, Spain; ^3^Division of Biostatistics, School of Public Health, University of Minnesota, Minneapolis, MN, United States

**Keywords:** geographical/spatial analysis, geostatistics, epidemiology, disease mapping, framework

## Abstract

Spatiotemporal visualization and analytical tools (SATs) are increasingly being applied to risk-based surveillance/monitoring of adverse health events affecting humans, animals, and ecosystems. Different disciplines use diverse SATs to address similar research questions. The juxtaposition of these diverse techniques provides a list of options for researchers who are new to population-level spatial eco-epidemiology. Here, we are conducting a narrative review to provide an overview of the multiple available SATs, and introducing a framework for choosing among them when addressing common research questions across disciplines. The framework is comprised of three stages: (a) pre-hypothesis testing stage, in which hypotheses regarding the spatial dependence of events are generated; (b) primary hypothesis testing stage, in which the existence of spatial dependence and patterns are tested; and (c) secondary-hypothesis testing and spatial modeling stage, in which predictions and inferences were made based on the identified spatial dependences and associated covariates. In this step-wise process, six key research questions are formulated, and the answers to those questions should lead researchers to select one or more methods from four broad categories of SATs: (T1) visualization and descriptive analysis; (T2) spatial/spatiotemporal dependence and pattern recognition; (T3) spatial smoothing and interpolation; and (T4) geographic correlation studies (i.e., spatial modeling and regression). The SATs described here include both those used for decades and also other relatively new tools. Through this framework review, we intend to facilitate the choice among available SATs and promote their interdisciplinary use to support improving human, animal, and ecosystem health.

## Spatial Epidemiology

Spatial epidemiology is defined as “the description and analysis of geographic variations in disease with respect to demographic, environmental, behavioral, socioeconomic, and infectious risk factors” (1). The importance of understanding the interplay between genetic, population, and environmental factors, and temporal characteristics of diseases in relation to space (2–4) has provided a set of powerful reasons to further develop the field of spatial epidemiology. The integration of epidemiological concepts, spatial analysis, geographic information system (GIS), and statistics leads to the accomplishment of the objectives of spatial epidemiology in understanding and modeling spatiotemporally explicit health risks (5–10). Essentially, geostatistics was originated in fields of geoscience, and the use of geostatistics on health data is synonymously referred to as “medical/health geography” or “spatial/geographical epidemiology” (11, 12).

The poster child of spatiotemporal epidemiological studies is Dr. John Snow's map of cholera deaths in Soho, London, in 1854 (13, 14). Dr. Snow used the map to support his theory that disease was associated with contaminated water, contrary to the popular belief at the time that it was airborne (14). Dr. Snow's classic work is an early example of how spatial epidemiological methods may support improving the quality of epidemiological investigations, eventually providing risk estimates in a timely manner to support decision and policy in preventive and control measures (15–17). Traditionally, spatial epidemiology focused on two major concepts: (a) mapping and spatial pattern analysis, such as cluster analysis, to determine visual and geographical relational cues (pre-hypothetical stages of research), and (b) using ecologic approaches to recognize etiologic clues of disease spread and explanatory factors (hypothesis-driven research) (18). However, the emergence of a large variety of tools and methods over the last decades has made the landscape of spatiotemporal epidemiological tools quite complex, challenging researches ability to identify the analytical approaches most suitable for their needs.

## Spatiotemporal Visualization and Analytical Tools (SATs)

A plethora of SATs, especially geostatistical tools, have been published and used in the field of spatial epidemiology (15, 19). However, for a beginner in spatial eco-epidemiology, selecting an appropriate analytical tool is often a challenging decision. Different disciplines, including epidemiology, econometrics, and ecology, use different SATs to address similar research questions (20–23). Juxtaposing these diverse techniques may support an interdisciplinary approach of shared knowledge while providing a list of options for researchers. The choice of SATs depends on a variety of factors/criteria. The majority of the published reviews and books on SATs are focused on describing the features of the tools/methods and do not guide a beginner researcher through the options to consider when choosing a spatial eco-epidemiological analysis. The objective of the paper here was to suggest a framework that facilitates choosing SATs which enables the researchers to analyze existing epidemiological data, draw inferences, and plan future research in spatiotemporal epidemiology.

## Data Used in Spatiotemporal Analysis

The types of spatial data that can be used in epidemiology to represent the distribution of diseases and adverse events in space include (1) point-referenced data (presence and absence of the disease or number of animals at each farm location), (2) point-pattern data (presence of the disease: where the disease occurrence itself is random giving rise to a “spatial point process”), and (3) areal data or “lattice data” (number of disease cases aggregated by an administrative division such as counties) (19, 24). The first case is often referred to as “geocoded” or “geostatistical” data (19). The point-referenced data and areal data may be of binary, count, or continuous in nature. The key difference between point-referenced and point-pattern data is that the former has a set of pre-known locations from which a certain value for a given variable was observed, whereas in the latter the events are assumed to have a stochastic or random nature (19). Therefore, in point-pattern data both the location and the observation of the disease themselves are random or stochastic. While the term “lattice data” may lead to the assumption that the areal units are regular shaped grids, in practice most areal data are summarized over irregular lattice such as administrative divisions. Reduced spatial explicitness may lead to aggregation of the events by administrative divisions and non-availability of the temporal details would limit the researcher to use purely spatial tools for the analysis.

While disease status data are the primary focus, epidemiological studies often look into association of the disease with underlying risk factors, such as human population density, air pollution parameters, temperature, precipitation, or soil pH among many other possible examples, which vary continuously over the space. These variables that are usable on GIS platforms are available from various data base sources in the form of point-referenced observations, polygon maps, or gridded i.e., “raster” maps. WorldClim [www.worldclim.org; (25, 26)] and LandScan Global Population Database (27) are examples of such data sources. The relevant value of these continuous variables, at each location where the disease status has been determined, can be extracted and used for further analysis, i.e., point-referenced data (19). The availability of exact location details and the time of the case supports more spatiotemporally explicit and reliable analysis. Unless specified as applicable to a particular type of data only, SATs described here are suitable to be used point-pattern, point-referenced, or areal data. It is important to notice that under certain circumstances the data types can be converted from one form to another. Point-referenced data can be summarized and represented by administrative divisions (i.e., polygon data). For example, point-referenced data representing 10 different farm locations recorded with a disease can be represented as 10 cases with in the county. Similarly, disaggregation of areal data with certain assumptions, such as density dependent disaggregation (28), is possible. Representing the area by the centroid of each polygon, thus, converting areal data into a point-referenced format, which, of course, is a simplification of the analysis that may be acceptable only under certain circumstances.

## A Framework for Choosing Spatiotemporal Epidemiological Tools

**Figure 1:**
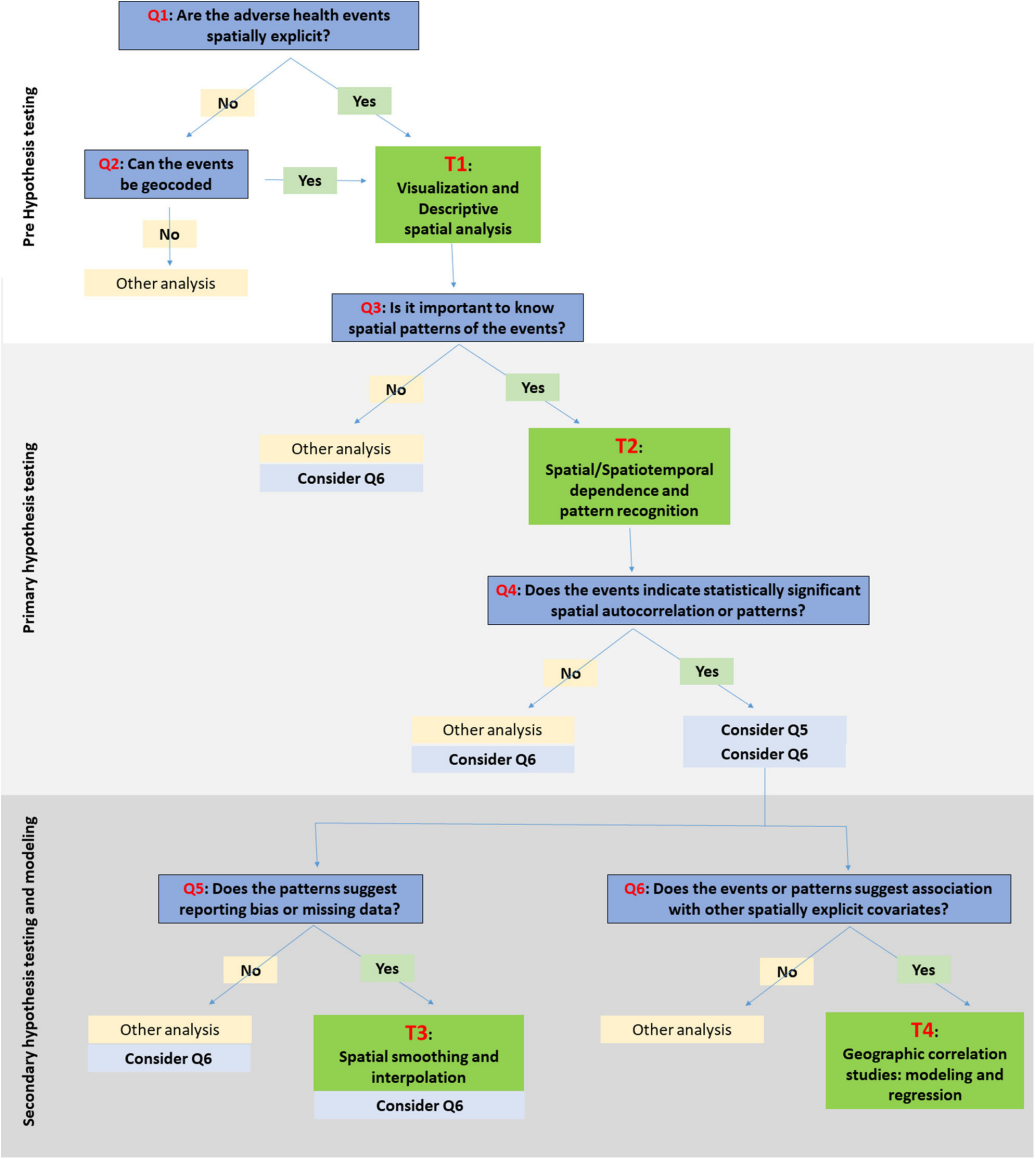
Schematic illustration of a framework for choosing spatiotemporal visualization and analytical tools (SATs). The research questions/objectives are identified with Q1:Q6. The specific SATs under the relevant categories, i.e., T1:T4, are listed in Table 1.

Here, we are suggesting a framework for choosing SATs (Figure 1). The framework is classified into three stages: (a) pre-hypothesis testing/hypothesis generating stage; (b) primary hypothesis testing stage; and (c) secondary-hypothesis testing and spatial modeling stage where the predictions and inferences are made. The primary hypothesis refers to the existence of spatial dependence and spatial patterns in the distribution of adverse health events, while the secondary hypotheses involve the association of the events with risk factors/covariates. The different types of SAT are broadly classified into four categories: (T1) visualization and descriptive analysis; (T2) spatial/Spatiotemporal dependence and pattern recognition; (T3) spatial smoothing and interpolation; and (T4) spatial correlation studies: modeling and regression. The types of data primarily applicable with different SATs are listed under T1:T4. The framework seeks to suggest a suitable category of the SAT among the four, based on the stage of the research question. The types of SAT that are commonly used in epidemiological studies are listed under each category (T1:T4) in Table 1 and discussed briefly below. The usage of tools are further discussed in relation to one example case study. It is important to note, however, that this is not a systematic review on the existing SATs, and that the classification used here is, somewhat, arbitrary, given the subjective nature of the problem. This contribution of a narrative review, while not an exhaustive description of SATs, intends to provide a short guide to introductory-level population and ecological scientists on commonly used tools and encourage the users to explore the diverse algorithms for more informed conclusions. Detailed reviews on SATs can be found elsewhere (6, 7, 10, 23, 138), as well as, a glossary of commonly used terms and their definitions in spatial epidemiology is found in Rezaeian et al. (11).

**Table 1:** A summary of types of common spatial analytical tools and their purpose.

	**Purpose**	**Measure**	**Commonly used techniques**	D*	**References**
T1: Visualization and descriptive analysis	Transformation of locational information into geographic coordinates	Geocoding/georeferencing	GIS based geocoding of street address, postal code, or administrative divisions	pp, pr, ar	(29–31)
T2: Spatial/Spatiotemporal dependence and pattern recognition	Visualization and description of the size and shape of the spatial distribution	Exploratory spatial data analysis	Mean center	pp, pr, ar	(32)
			Median center		(32)
			Convex hull		(33)
			Standard deviation (weighted by attributes)		(32)
			Directional mean and variance		(34)
			Moran scatter plot		(35)
		Characterize nearby features	Features with in a distance band/buffer zone	pr, ar	(31, 36)
			Distance to feature		(31)
			Overlaying features		(31)
	Test whether there is spatial dependence in the event data	Spatial autocorrelation	Global Moran's I	pr, ar	(37)
			Geary's C		(38)
			Mantel test		(39)
			Geti's ord		(40, 41)
		Spatial autocorrelation among regression residuals	Moran's I test	pr, ar	(42, 43)
			Kelejian–Robinson test		(44, 45)
		Distance analysis	Nearest neighbor analysis		(46)
			Ripley's K		(47, 48)
			Distance matrices		(31)
	Measure the uneven distribution of the populations and risk factors	Local or stratified spatial heterogeneity	Getis Ord Gi*	pr, ar	(40, 41)
			K-means clustering		(49)
			Anselin's local Moran's I (L-Moran)		(50)
			Spatial stratified heterogeneity test		(51)
	Measure the spatial dependence while accounting for background population		Oden's Ipop	ar	[(52, 53); https://www.biomedware.com]
	Test whether there is any spatial trends	Testing for first-order effects	Trend analysis	pr, ar	(18, 54, 55)
	Test whether there is any spatial clustering in the data	Global cluster detection	Nearest neighbor test	pp, pr, ar	(46)
			Cuzick and Edward's test (case-control data)		(56)
			Local indicators of spatial association (LISA)		(50)
	Locate the clusters and the statistical significance of the clustering	Purely spatial local cluster detection	Spatial scan statistics Flexscan	ar	(57–59) (60)
			Turnbull's test	pr, ar	(61)
			Besag and Newell's test		(62)
	Test whether there is space and time clustering in the data	Spatiotemporal cluster detection	Knox test	pp, pr, ar	(63)
			Mantel test		(39)
			Barton's test		(64)
			kth nearest neighbor test for time-space interaction		(65)
			Space-time permutation scan statistic		(66, 67)
			Edrer-Myers-Mantel test		(68, 69)
	Detect the direction of progression of an event over time	Spatiotemporal directionality	Spatiotemporal directionality test	pr, ar	[(53, 70); https://www.biomedware.com]
			Spatiotemporal anisotropy parameter		(71, 72)
T3: Spatial smoothing and interpolation	Quantifying spatial variations in event intensity: spatial point pattern (SPP) intensity	Density based point pattern recognition	Univariate Kernel density estimation (KDE)	pr	(73–75)
			Multidimensional KDE		(76, 77)
			Empirical Bayes smoothing (EBS)	ar	(78, 79)
	Smoothing and interpolation	Deterministic spatial interpolation	Thiessen (Voronoi) polygons	pr	(80)
			Neighborhood matrices		(31)
			Inverse Distance Estimation (IDW)		(32, 81, 82)
			Triangulated Irregular Network (TIN)		(83, 84)
			Headbang smoothing		(85–87)
		Spatial modeling with stochastic partial differential equations (SPDE)		pr	(88, 89)
		Geostatistical interpolation and spatial regression	Kriging	pr	(32, 90, 91) (92) (93–96)
			Spline regression models		
			Trend Surface Interpolation		
		Multivariate spatial interpolation	Co-kriging	pr	(32, 91, 97) (98–100)
			Regression kriging		
		Spatiotemporal interpolation	Space-time kriging	pr	(101, 102) (103)
			Autoregressive spatial smoothing and temporal Spline smoothing		
T4: Geographic correlation studies: modeling and regression	Estimate the probability of disease spread using explanatory variables	Regression at spatial units	Ordinary least square regression and test for spatial autocorrelation of residuals	pp, pr, ar	(42, 43, 45)
			Spatial lag model with independent variable representing neighbors		(104, 105)
		Spatial and spatiotemporal error autoregression models for areal data (When regression residuals have spatial autocorrelation)	Simultaneous autoregressive (SAR) models	pr, ar	(19, 24, 106)
			Geographically weighted regression (GWR)		(107, 108)
			Purely spatial: Conditional autoregressive (CAR) models		(19, 109, 110)
			Spatiotemporal CAR models		(111, 112)
			Two-stage space-time mixture modeling		(113)
			Latent structure models		(113–115)
		Spatial and spatiotemporal models for point-level data	Point process models with weighted sum approximation	pp	(116, 117)
			Conditional logistic model	pp, pr	(118, 119)
			Separable models for spatiotemporal data		(19)
			Non-separable models for spatiotemporal		(19)
	Measure the gravitation of adverse effects and the risk factors based on distance	Estimate most probable spatial interactions between entities	Gravity models	pr, ar	(120–123)
	Analysis of spatially explicit time-to-event data	Spatial survival models	Spatial cure rate model	pr	(124)
			Frailty models		(124)
	Estimate the probability of disease when the disease occurrence is correlated with environmental variables	Environmental/Ecological niche modeling	Maximum Entropy Ecological Niche modeling (Maxent)	pr	(125–127)
			Genetic Algorithm for Rule Set Production (GARP)		(128–130)
		Machine/statistical learning techniques	Random forest	pr	(131, 132)
			Generalized additive models (GAMs)		(133–135)
			Artificial neural networks (ANN)		(136, 137)

D* Column represents the type of data primarily applicable on the set of tools, where, pp, point-pattern; pr, point-referenced; ar, areal data.

## Commonly Used Spatiotemporal Visualization and Analytical Tools (SATs)

### T1 Tools for Visualization and Descriptive Analysis

Spatial data visualization is one of the key steps in understanding and generating hypotheses on the spatial distribution of events. Global Navigation Satellite Systems (GNSS), such as Global Positioning System (GPS); Global Navigation Satellite System (GLONASS); Galileo; Navigation Indian Constellation (NavIC); and BeiDou provide the ability to position the exact geospatial locations during the data collection phase. In the absence of GNSS based data, geocoding plays a major role to generate spatially explicit databases (29, 30). In addition to the visualization, description of the extent of spatial distribution by means of size, shape, and directionality of the spread supports understanding the extent of the adverse health/environmental effect. Descriptive analysis using T1 tools may support planning primary interventions including assigning vaccine or surveillance buffer zones and recognizing the distance to closest epidemiologically important features.

GIS is a system which enables capturing, storing, visualizing, and analyzing spatially explicit or “georeferenced” data to cartographic projections (31, 139). The true value of the ability to place data or measurements on a map, either as discrete events using its exact location (i.e., point-referenced data) or as continuous data by regular grids (i.e., raster data), is the ability to assess possible relationships within the data. GIS technology makes it technically feasible to integrate large amounts of data collected from different sources into a single georeferenced map/model for analysis. Therefore, GIS plays a major role in the spatial analysis as a platform which facilitates bringing data and analytical techniques together. The key analytical tools are listed under T2:T4.

### T2 Tools for Spatial/Spatiotemporal Dependence and Pattern Recognition

#### Measures of Spatial Autocorrelation

According to Walter Tobler's First Law of Geography, “everything is related to everything else, but near things are more related than distant things (140).” This phenomenon, otherwise known as spatial autocorrelation or spatial dependence, is a key component of spatial epidemiology. The majority of the T2 techniques are focused on determining the extent to which data are spatially autocorrelated and performing hypothesis tests after accounting for spatial autocorrelation (141). Assumptions involved in the analytics include the spatial stationarity, isotropic spatial autocorrelation, and spatial continuity (141). In simpler terms these assumptions imply that events (infectious diseases in animals for example) of the considered spatial process are homogeneously distributed across the region regardless of geographical directions or barriers. However, understanding the violations of these assumptions, i.e., detecting patterns of non-stationarity or anisotropy, is paired with the descriptive analytics (32). Moran's I (37), Geary's C (38), Mantel test (39), and Getis Ord (40, 41), which often referred to as “global spatial autocorrelation indices” (142) are the commonly used techniques to measure spatial autocorrelation.

Measurement of spatial heterogeneity, i.e., uneven distribution of the populations and risk factors across the geographical space, is another important component for understanding the disease process. Spatial heterogeneity measures could be either (1) local where we measure whether an attribute at one site is different from its surrounding or (2) stratified where the attributes are stratified within strata, such as Agro-ecological zones or land use categories in which the spatial variance between strata was measured. An example of local measures of spatial heterogeneity is Getis Ord Gi^*^ [i.e., hot-pot/cold spot analysis; (40, 41)]. Other techniques such as G-statistics are increasingly available facilitating the measurement of stratified spatial heterogeneity (51). The indices of spatial heterogeneity provide opportunity to quantitatively measure the differences and compare the landscape patterns of populations and risk factors.

#### Spatial Cluster Analysis

A spatial cluster is an excess of events or measurements in certain areas in geographic space, compared to the null expectation of complete spatial randomness (143). The cluster analysis is generally aimed at detecting if there is any clustering in the spatial data (i.e., Global cluster analysis), and detecting and locating the clusters (local cluster analysis and focused cluster analysis). In general, the cluster analysis provides information about the cluster morphology, including the magnitude of the excess/deficit feature, geographic size, shape, and the locations of spatial clusters.

Detecting first-order adjacencies such as Local Indicators of Spatial Autocorrelation (LISA) statistics (41, 50) and nearest-neighbors relationships such as used in Cuzick and Edward's (56) test can be considered as global cluster detection techniques. Most local cluster-detection techniques employ circular scanning windows, such as the scan statistic (58), Turnbull's test (61), and Besag and Newell's (62) test. In scan statistics, a circular scanning window of varying sizes that moves across the study area is used to compare the observed-to-expected ratio of the cases compared to the expected spatial randomness was calculated, and the windows that maximize this likelihood ratio were recognized as the most likely clusters (58). Some of these local cluster analyses such as scan statistics have been incorporated into widely used software such as SaTScan that enable temporal, spatial, and spatiotemporal cluster analysis in a user-friendly manner. However, it is essential to realize that spatial variation and hence cluster morphology is complex, and may not be well-described by the circular cluster window approaches (143, 144). Therefore, alternative approaches that are flexible for the cluster shape such as Flex scan (60), Upper Level Set scan statistics (145), and B-statistics (146) have been introduced. A detailed description on the spatial pattern recognition and cluster analytical techniques are found elsewhere (143). The performance of SATs designed to detect clusters can be highly sensitive to the level of aggregation of the data (147). Therefore, while the clusters detected based on point-pattern or point-referenced data are intuitive to interpret, the clusters of data aggregated at large areal units requires caution. Distance based assignment of the neighbors instead of considering shared borders between areal units has been suggested (147). Morris and Munasinghe (148) have offered a solution through a user defined computer algorithm that combines existing areal units, such as administrative divisions, into regions with populations large enough to diminish spurious variability in disease rates while limiting the loss in resolution.

### T3 Tools for Spatial Smoothing and Interpolation

#### Spatial Smoothing Techniques

Many research studies on adverse health/environmental events apply spatial smoothing and interpolation techniques to improve estimation and for exploratory mapping of risk (149). There is a variety of smoothing techniques and they can be broadly categorized as global (the same function is applied to all the data points and predictions are made using the entire dataset) and local (the same function is applied to sub-sets of data points based on the neighborhood) smoothing techniques. Kernel smoothing, one of the widely used techniques, facilitates visualization of the intensity of events (73) while accounting for background spatial distribution of the population at risk (150), and generate tolerance contours (i.e., confidence regions) for which the relative risk of a disease is significantly high (74, 75). Kernel smoothing can be used to describe and visualize the intensity or the spatial relative risk of health threats. Smoothing techniques are used to reduce noise by shrinking values toward the adjacent observations and estimate the spatial trend, which is applicable to both homogenous and heterogeneous point processes (75, 151). In a heterogeneous point process in which the intensity of the spatially varying event varies within the study area, smoothing is used to increase accuracy of the estimation of the event intensity using either parametric or non-parametric methods (73–75). Spatial smoothing techniques use a moving weighted function to reduce the noise component, where the differences in the values on a surface are accentuated resulting in a spatially continuous map. Commonly used spatial smoothing techniques include kernel density estimation (KDE) [(73, 74, 152, 153)] and headbanging (85–87), which are considered as alternatives of detecting circumscribing clusters of varying shapes in lieu of circular clusters (74, 143). Empirical Bayes smoothing (EBS) is a specific case of spatial smoothing where the denominator i.e., varying population at risk over the map is used as a measure of the confidence in risk estimates. Therefore, the confidence of estimates are higher in highly populated areas, whereas, the estimates of relative risk would have high margins of error in the less populated areas (79). For example, if two counties have same the standardized incidence ratio (SIR) but have different population sizes, the confidence of EBS estimates would be higher for the county with a larger population size.

#### Spatial Interpolation Techniques

Spatial interpolation techniques are used to estimate or predict values at unknown locations using available/known data points (32). These tools can be broadly categorized as deterministic (they use the extent of similarity or distance to create the surface using measured points) and geostatistical (they use the statistical properties of the measured points to create the interpolated surface) interpolations. The resulting interpolated surfaces i.e., statistical surfaces are raster layers and often can be considered as risk maps in epidemiological analyses. There are multiple spatial interpolation techniques including Inverse distance estimation (IDW) (81), Triangulated Irregular Network (TIN) (5, 83), Kriging as well as its variations such as Co-kriging (32), and Trend Surface Interpolation (93–96) are among the commonly used techniques. TIN represents the surface by a set of contiguous and non-overlapping triangles connecting the original data points and allows construction of 3-dimensional surfaces based on a secondary variable of a researcher's choice, which, for example, the prevalence of a disease in a farm location. A review by Li and Heap (84) summarizes and compares several interpolation methods used in environmental sciences that are highly applicable in eco-epidemiological studies as well.

Geostatistical interpolation, such as kriging can be understood as a two-step process, where, step 1 is fitting the spatial variogram or likelihood for the data observed at the sampled points; and step 2 involves the interpolation of values for unsampled points or blocks using the weights derived from this covariance structure (32). In situations in which disease events are biased or undersampled, co-kriging can be used to enhance the accuracy of the estimation using a highly sampled auxiliary variable (154). For example, when invasive species detected at lakes are underreported, but the known invasions are highly correlated with the visitors/boater traffic in-and-out of the lakes and data are available for this variable, boater traffic network may use as an auxiliary variable to determine the lakes that are likely to be invaded (155). Trend surface interpolation facilitates mapping variables while allowing for the local fluctuations. Therefore, trend surface analysis may reflect the regional distribution, trend, and the local variation of the mapped disease (156, 157). Interpolation techniques, their model assumptions, and usage are discussed extensively, elsewhere (32, 96).

Spatiotemporal interpolation techniques are used to predict variables in-between and beyond observation times (101, 102). In space-time kriging, the spatial, temporal, and spatiotemporal dependence structures are modeled using spatiotemporal variograms (102). Modeling the spatial and temporal components independently is one of the drawbacks in most of the spatiotemporal interpolation techniques (158). A detailed discussion on the spatiotemporal interpolation techniques used in the environmental modeling is found elsewhere (158). Recent developments including spatial modeling with stochastic partial differential equations (SPDE) have further improved spatial and spatiotemporal smoothing using Bayesian inference (88, 89).

### T4 Tools for Geographical Correlation Studies: Modeling and Regression

#### Spatial Regression Models

In geographic correlation studies in epidemiology, spatial regression analysis is commonly used to examine the effects of certain risk factors/covariates on disease incidence while accounting for the spatial autocorrelation/dependence (19, 104, 159–161). Spatial dependence is incorporated into the model specifications typically using a spatial lag term or spatial error autorregression models [i.e., assigning autoregression terms for regression residuals; (104, 160)]. This is because the standard regression models assume that observations are independent, an assumption that is not met when spatially dependent data are analyzed. Fitting regression models while assigning a variable to represent the neighbor effect is one way of modeling the spatial dependence. For example in spatial lag model in which we assume that disease status in at one location is affected by the disease status at the nearby locations, a “lag” term, which is a specification of disease status at nearby locations, is included in the regression, and its coefficient and *p*-value are interpreted as for the independent variables (104). Both Frequentist and Bayesian spatial regression techniques have been extensively used in epidemiological analyses. Spatial regression models vary by their computational complexity, capacity of capturing spatial heterogeneity, and the quantification of uncertainty associated with parameter estimates (161).

Spatial error autoregressive models for discrete/areal data include: Simultaneous autoregressive (SAR) models (19, 24, 106, 162), Geographically weighted regression (163), and Conditional autoregressive models (CAR) with neighborhood structures defined based on Besag, York, and Mollie (BYM) model or Leurox (109, 110). Defining the neighbors for areal data is done based on contiguity including first-order contiguity (i.e., presence of shared borders between polygons such as adjacent counties); graph-based contiguity (i.e., based on defined algorithms such as nearest-neighbor graphs); or distance-based contiguity [i.e., neighbors within 10 km; (45)]. Due to sampling and reporting variabilities of disease incidences and risk factors, borrowing strength from neighboring regions to get more reliable estimates is the motivation behind these spatially dependent regression models (e.g., closer neighbors might receive higher weights). This strategy of borrowing information from neighbors is applicable in autoregressive models, where the spatial or spatiotemporal structure is modeled via sets of autocorrelated random effects (19, 109, 164).

In addition to accounting for the spatial dependency, multiple spatiotemporal regression models have been used in epidemiological studies that enable the researchers to analyze the influence of spatial and temporal dependence of disease events and risk factors (19, 165). Detailed descriptions on spatial and spatiotemporal autoregressive models can be found elsewhere (19, 165). For example, latent structure models which accounts for the heterogeneity or the discontinuity in risk surface such that homogenous areas can be grouped together while discriminating for the risk levels (114).

When the events are recorded as point-referenced data from locations within a continuous spatial domain, such as by households or animal farms in a certain area, the binary outcome that the adverse event occurs in each location is assumed to have an underlying continuous spatial process. Spatial processes with binary outcomes are usually modeled by spatial logistic or probit regression models. Assigning the spatial dependence and neighbors in spatial process is complicated. This is because point-referenced spatial data often come as multivariate measurements at each location and we anticipate dependence between measurements both at a particular location as well as across locations. For example presence of a certain animal disease in a farm is correlated with the farms own characteristics including number of animals and management practices, as well as the presence of neighboring farms. Separable and non-separable spatiotemporal regression models are commonly used to model spatial point processes (19, 166).

#### Environmental/Ecological Models

Ecological niche modeling (ENM) approaches are widely used to characterize the complexity and heterogeneity of the landscapes in research related to epidemiologically relevant vector and parasite-reservoir distributions (167, 168). In addition to the characterization of the areas where disease is distributed, ENM is used to identify potential distributional areas in response to the likely geographic shifts in distributional areas of species or phenomena under scenarios of climate change or changing land use (169). Genetic Algorithm for Rule Set Production (GARP) (129, 130); Maximum Entropy Ecological Niche modeling (Maxent) (125, 126); and Machine/statistical Learning Techniques such as random forest (131, 132) and artificial neural networks (ANN) (136, 137) are the commonly used algorithms in epidemiology. Most ENM studies use presence-only data for the analyses. Further details regarding GARP, Maxent, and other ENM algorithms are found elsewhere [(125, 126, 128, 129)]. Additionally, hybrid methods that are bringing together multiple tools are being used in several disciplines to improve estimation and prediction abilities in spatial analysis.

## Evaluating the Performance of Spatiotemporal Analytical Tools

### Model Performance Indicators

Evaluating model performance is important when choosing between similar SATs (Especially those listed under T3 and T4). These measures include correct classification rate (CCR) (170), model sensitivity and specificity (i.e., the number of correctly classified cases) and area under the receiver operating characteristics (ROC) curve (170, 171). The sensitivity of a spatial model in disease mapping can be defined as the model's ability to correctly predict high-risk areas/locations, whereas, the specificity of the model would be its ability to correctly identify low-risk areas/locations. Error and accuracy measures, such as root mean squared error (RMSE), are also used to measure how wrong the resultant model estimates can be (138). Similarly, penalized-likelihood criteria for comparing models including Akaike information criterion (AIC) (172), Bayesian information criterion (BIC) (173), Deviance information criterio (DIC) (174, 175), and Watanabe-Akaike information criterion (WAIC) (176) are used in regression models as relative measures to compare between models and evaluate goodness of fit with penalty on model complexity. Further reading on the choice of model selection criterion is found elsewhere (177, 178).

### Model Validation Techniques

The SATs, especially the predictive modeling-and correlation models (listed under T3 and T4 of Table 1), are evaluated for their performance because the predictions would have no merit if the accuracy of the models cannot be assessed using independent data (138, 170, 179). A variety of techniques are available to validate the SATs (Listed under T3 and T4 of Table 1). Data partitioning techniques such as bootstrapping (180, 181), randomization (182), prospective sampling (182, 183), and k-fold partitioning (184, 185), leave-one-out cross-validation (138) are commonly used to determine training and testing datasets for model validations.

Cross validation, i.e., partitioning the data into several subsets and each fitting the model excluding one subset and validating the fitted model's ability to correctly predict the risk areas using the excluded subset of data, is one of the common practices in spatial model validation (138, 185). This includes dividing the data over space or time. For example, if the incident data are from 2000 through 2018, fitting model using early data/incidents and validation of the model predictions using recent events is considered an approach of temporal cross validation. Temporal cross validation is also achieved through the prospective sampling where new cases are evaluated against already built models from a different region or from a different time (170). A review by Anselin (179) discuss model validation techniques used in spatial econometrics in relation to the statistical validity of the models. The model fitting concerns related to theory, hypothesis testing, choice of criteria, and practical considerations are discussed under this criteria of model validations (179).

## Available Software Tools Facilitating SAT

Multiple free and proprietary software tools are available facilitating the spatiotemporal analytical studies. However, there is no quality control over to assess the accuracy, reliability, and sustainability of the majority of those non-proprietary software. Some software, such as SaTScan^TM^ (https://www.satscan.org) and ArcGIS (https://geocode.arcgis.com), have become successful commercial products that are widely in use (7, 186), while others are underutilized due to less popularity and irregular maintenance. Sustainability and maintenance of these software is essential when incorporating these software based eco-epidemiological analyses into surveillance or intervention measures. An overview of the spatial data analytical software is found elsewhere (186).

Geocoding can be implemented using either commercial GIS software or online that are developed by governmental (Ex. USGS map locator: https://store.usgs.gov/map-locator), private (ArcGIS Online Geocoding Service by Esri (https://geocode.arcgis.com/arcgis/); QGIS Geocoding Plugins (https://plugins.qgis.org/plugins/GeoCoding/); Geocoding using Google maps (https://cloud.google.com/maps-platform), or through educational organizations (e.g., TAMU Geo coding Services of the University of Texas A&M: http://geoservices.tamu.edu/). Similarly, Python based geocoding using open or commercial spatial data repositories and spatial database management systems such as Google geocoding application programming interface (API) and improving the capacity of spatial computing is a field in developing (187). These software and tools enable both batch geocoding where multiple addresses are submitted at once for geocoding, and reverse geocoding, i.e., determining the nearest street address based on given coordinates.

The commonly used user-friendly software in the spatiotemporal analysis that are capable of performing the descriptive analysis, spatial pattern recognition, smoothing/interpolation, and/or spatial modeling are ArcGIS (188), QGIS (189), GRASS (190), GeoDa [(191); http://geodacenter.github.io/index.html], Clusterseer [(53); https://www.biomedware.com/], SaTScan (http://www.satscan.org/version 9.6), and CrimeStat (192). Similarly, there are multiple toolboxes relevant to spatiotemporal analysis that can be used through following software: R statistical software (193), SAS (194) (SAS/STAT® software), STATA (195), and Matlab (Matlab: https://www.mathworks.com)^1^. platforms that are specifically developed for handling geospatial analysis. Some of the advanced statistical software packages enables performing both frequentist and Bayesian spatial analyses. For example, the R package “spatialreg” (196, 197) enables performing frequentist spatial error models including CAR models (listed under T4), while R packages “CARBayes” (198), “CARBayesST” (165), and “R-INLA” [(88); www.r-inla.org; (199)] enables fitting Bayesian CAR models using Markov Chain Monte Carlo (MCMC) or Integrated Nested Laplace approximation (INLA) based estimation of the posterior distributions, respectively.

## How to Use the Framework to Choose SAT: An Example

While we have introduced a framework and a categorization of commonly used SATS, it is important to note that the choice of the SATs is entirely a researcher-driven decision. There are certain factors/criteria associated with the decision of choosing one method over the other. The factors include: (1) characteristics of the disease/adverse event; (2) study design; (3) spatial explicitness of data; (4) data quality and availability; (5) research question and hypothesis; (6) stakeholder involvement; and (7) existence of resources, policy, and regulations for the mitigation of events (200). These factors influences the six questions (Q1:Q6) illustrated in the framework (Figure 1).

For example, assume a researcher is interested in understanding epidemiological characteristics of natural Anthrax in animal populations and intends to use that information to plan a surveillance/vaccination program in an endemic area. Let us assume that the final output the researcher intends to have is a criteria to define zoning distances for ring vaccination or surveillance when at least one Anthrax case is reported. Firstly, understanding the extent of spread and duration of previous Anthrax outbreaks would play a major role when determining this surveillance/vaccination radii. Secondly, understanding the association between the epidemiological drivers of the disease and the characteristics of susceptible population would be of importance when planning an area-based surveillance/vaccination program.

At the pre-hypothesis stage of the framework (Supplementary Figure 1), answering questions Q1 and Q2 would guide the researcher to use T1 tools and obtain a spatially explicit data set that is ready for further spatial analysis. Anthrax, caused by a spore-forming bacterium *Bacillus anthracis*, is characterized by the prolonged survival of the spores on soil and wide range of hosts including wildlife, livestock, and human (201, 202). Therefore, the observational study designs on Anthrax are likely to be retrospective based on reported cases (203). Given Anthrax is reportable to the animal and public health authorities, most likely type of data available would be point-referenced in nature (i.e., presence of the disease at farm locations or grazing lands). Although in rare situations, data may be available aggregated at administrative divisions due to privacy policy. If the coordinates of case locations are not recorded along with the case report, geocoding the locations based on the descriptions or farm addresses would be the initiating step.

Once geocoded, answering the Q3 and the use of SATs listed under T2 would facilitate the recognition of spatiotemporal dependence between the reported cases (i.e., the primary hypothesis testing stage). Given the prolonged survival of Anthrax spores in contaminated soils/environment, in addition to the initial testing for spatial dependence, understanding the spatiotemporal dependence and spatiotemporal directionality is the key to understand the extent of past spread of the disease. Testing whether there are space and time clustering in the data would facilitate determining any particular area/s with high relative risk for disease clusters at a specific time [i.e., disease hot-spots; (203)].

Once geocoded, the primary hypothesis testing stage of the framework and the T2 tools would facilitate the recognition of spatiotemporal dependence between the reported cases and determining any particular area with high relative risk for disease clusters [i.e., disease hot-spots; (203)]. Given the prolonged survival of the Anthrax spores conducting purely spatial and spatiotemporal dependence and directionality is the key to understand the extent of past spread of the disease. This spatiotemporal pattern detection may lead to the refinement of further research questions (Q4: Q6 of the framework) and secondary hypothesis testing using the SATs listed under T3 and T4 (Supplementary Figure 1).

Because the pathogen is invariably dependent upon the distribution of susceptible species and environmental characteristics such as soil pH, rain fall, and flood plains; the choice of predictive modeling using correlated environmental factors such as regression or ecological niche modeling (ENM) (204) is a suitable option to consider (i.e., tools under T4). However, it is important to recognize that the ideal analysis for a chronic disease like Anthrax would be spatiotemporal correlation models that enable incorporating temporal changes of both the disease and underlying environmental characteristics, in addition to space.

Once the range of cluster radii (T2 tools) and key epidemiologically important environmental factors by area (T4 tools) were identified, these two key pieces of information would facilitate informing the decisions of planning the ring vaccination/surveillance programs. For example, recognition of which areas are at high risk for Anthrax based on the models outputs from T4 tools, such as ENM (204), and the extent/cluster radii of past outbreaks using T2 tools would allow us to inform defining the minimum and maximum zoning distances for ring vaccination/surveillance.

## Advantage, Challenges, and Drawbacks of SATs

The framework provides an introductory guide for choosing SATs for eco-epidemiological studies. Use of SATs improves an eco-epidemiological investigation by adding precision, facilitating the comparison of distributions by means of quantitative criteria, and capturing risk factors and characteristics that are unlikely to be detected by visual inspection or analyzing data without the spatial component (6). Therefore, SAT outcomes, commonly represented as “risk maps,” may serve as estimates of the effects of “real” exposures to human, animal, and environmental health threats and facilitate recognizing the effect size at more vulnerable locations and time periods.

Common weaknesses associated with the spatial analysis and risk mapping are related to shortcomings in the accuracy of data, choices of mapping and projections, choice of the analytical/ modeling tools and relevant assumptions, and eventually the decisions related to the representation of the risk maps to the end users (205, 206). In relation to the data aggregated by administrative divisions, commonly discussed issues include “edge effect” i.e., problems posed by the presence of adjacent locations not included in the analysis but that can influence its outcome, such as an unknown disease status in a country adjacent to the study area [(207, 208)]; and the “modifiable areal unit problem (MAUP)” i.e., the existence of differences in the analytical results obtained through the analysis of the same input data after aggregation at different levels. Examples include aggregation of point data from dairy farms in to counties or data available at sub districts level into provinces. The MAUP pertains to scale and zoning effect of the divisions (209, 210). A variety of methods are discussed in the literature to quantify and account for the edge effect and MAUP issues (211, 212). When spatial analytics and models are conducted based on available and potentially biased data, the resulting risk maps are invariably subjected to the negative impact of the data quality. However, we emphasize the use of existing data, bringing several databases together, and the spatiotemporal analytical tools can support initiating the process of improving data quality.

The choice of SAT, as discussed, varies with multiple factors. Inevitably, all analytical tools and models involve certain assumptions on statistical properties of variables and often these assumptions are violated in natural environments. In other words, none of the SAT are perfect matches for any particular situation (158). For example, spatial continuity of risk is a common assumption in risk-mapping process while there can be natural (e.g., mountain range acting as a physical barrier) or infrastructural barriers (e.g., urban vs. rural neighborhoods) that violate the continuity assumption resulting in step changes of risk between adjacent areas (112). Therefore, clarity on the choice of SAT, underlying assumptions, and the seven factors/criteria is essential when choosing SAT to address eco-epidemiological problems.

## Future Directions

Improving the quality of spatially explicit health and environmental data through systematic collection of high-resolution data and public participation GIS approaches such as “crowdsourcing” or “citizen science data” is increasingly popular in both public and environmental health monitoring efforts (213–215). Additionally, the use of existing databases as passive surveillance systems and improving systematic data collection are suggested as ways to generate spatially explicit animal health databases (203).

While the geostatistical techniques introduced here, especially those under T4, commonly are frequentist approaches. The hierarchical specification of geostatistical models (216), therefore the adoption of a Bayesian framework for inference and suitable Gibbs sampling, MCMC, or INLA [(88); www.r-inla.org; (199)] for model fitting is being increasingly used. In addition to the geostatistical SATs discussed here, there are non-geostatistical spatial analytical tools such as Agent-based modeling (217–219) that are increasingly used by the researchers interested in spatial eco-epidemiological studies.

When modeling complex systems of adverse health and environmental effects, incorporation of several other analytical and modeling techniques in addition to SATs may support further exploring the phenomena including understanding the network effects (21). Spatial networks are another branch of the complex system approaches to spatial data. Because complex systems are often organized under the form of networks where nodes and edges are embedded in space, such as transportation networks of swine farms or water connectivity networks between salmon farms, the importance of connectivity in addition to the spatial proximity has a major role when determining disease transmission (220).

Predicting where the phenomenon would move/flow/spread next is an essential component in spatial modeling. SATs such as space-time kriging (T3 of Table 1) are capable of estimating such phenomena (221). Atmospheric dispersion models such as plume models (222) and Hybrid Single Particle Lagrangian Integrated Trajectory Model (HYSPLIT) (223) are examples of applications of spatial models that account for flow directions and cost surfaces used to predict wind-mediated transmission of arthropod-borne diseases. While these models can be considered as advanced spatiotemporal variations of SATs listed under T4 here, they can be computationally costly. Hence, for the researchers who are new to population-level spatial analysis and models, it is recommendable to start with the simpler and more established SATs to explore health or environmental threats prior to applying novel modeling techniques.

## Author Contributions

KK designed the framework, directed the review process, and wrote the article. JA and LZ provided expertise in methods and edited and reviewed the manuscript. AP contributed in design, expertise in methods, supervision, and revision of the manuscript. All authors contributed to the article and approved the submitted version.

## Conflict of Interest

The authors declare that the research was conducted in the absence of any commercial or financial relationships that could be construed as a potential conflict of interest.

## Footnotes

^1^*MATLAB* and Statistics Toolbox TM Release 2018a. Natick, MA: The MathWorks, Inc.
