# Supplementary figures and images for: An Introductory Framework for Choosing Spatiotemporal Analytical Tools in Population-Level Eco-Epidemiological Research
Source: Front Vet Sci. 2020 Jul 7;7:339. doi: 10.3389/fvets.2020.00339 (PMC7358365; doi:10.3389/fvets.2020.00339)

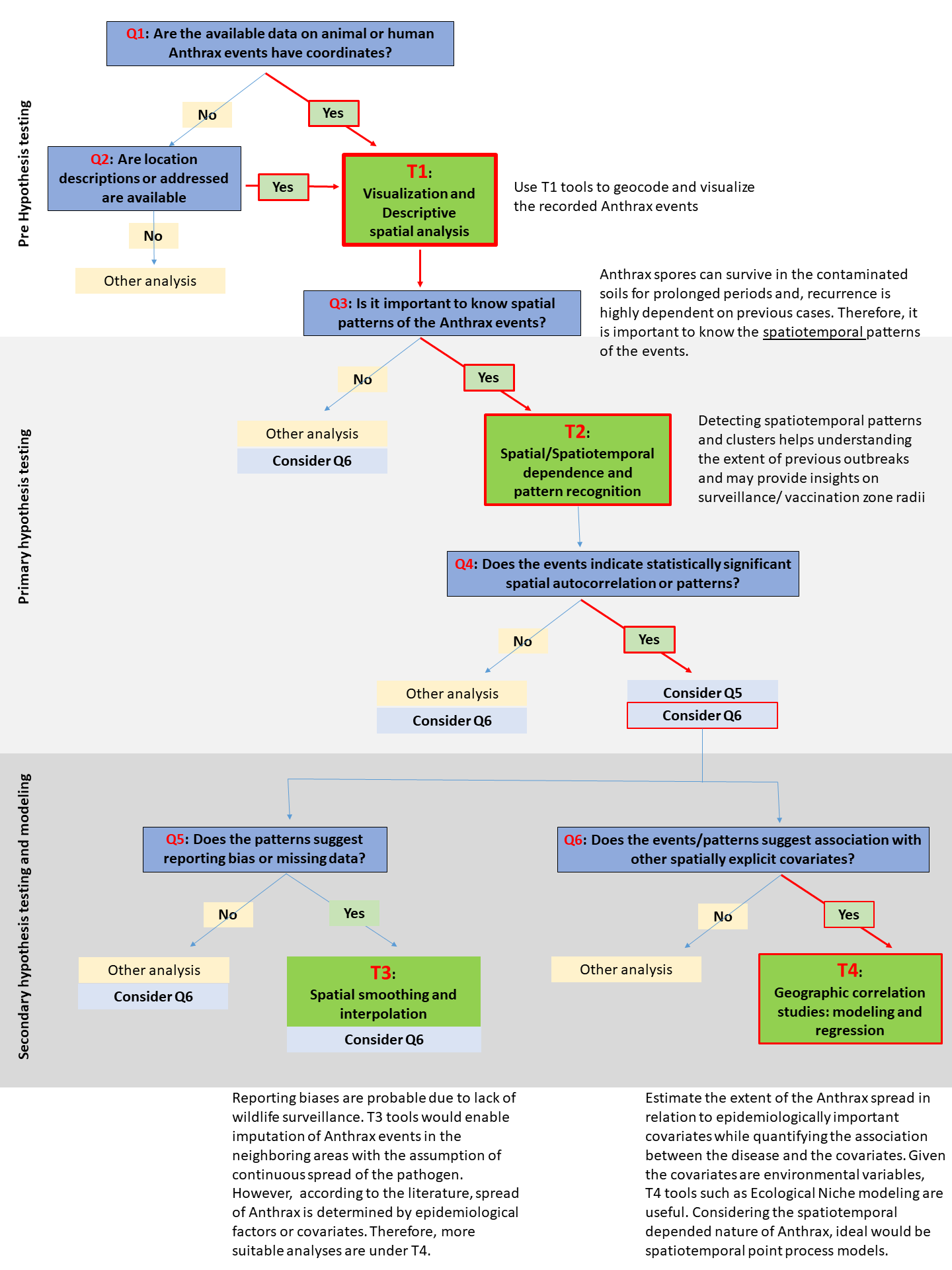

Supplement: Supplementary file 1 [file Image_1.TIF]
